# Biographical Sketch of Dr. Alvin Blakesley, of Utica. N. Y

**Published:** 1860-11

**Authors:** 


### BIOGRAPHICAL SKETCH OF DR. ALVIN BLAKESLEY, OF UTICA, N. Y.

In presenting the following remarks upon the life and history of Dr. Blakesley it is not without a due sense of the difficulty attending such a work, that we attempt it.

In attempting to give a portraiture of characteristics, the only proper course is to adhere faithfully to the original; and especially is this the case when the subject is one who has occupied a prominent position. It was only during the last three or four years of his life that we were personally acquainted with Dr. B. But for twelve or fifteen years we had known him by reputation, and our knowledge of him warrants the assertion that he was a man of sterling worth in his profession, in society, and especially in his family. Not quite two years ago, he made the following remark, "I am the happiest man in the world in my family relations."

We can present but a brief sketch of his early life, and for this we are indebted chiefly to Dr. Swartwout of Utica, N. Y., the place of Dr. B.'s late residence.

He was born in Trenton, Oneida Co., N. Y., Aug. 19, 1798. His parents moved when quite young to Vienna, where he lived most of the time with his grandfather; his educational advantages in early life were very limited, having attended only those schools common in country villages in those days. A portion of his time was spent in agricultural pursuits, but being of too feeble constitution for that occupation, he went to Clinton, Oneida Co., at the age of eighteen years, to learn the trade of watch-making, in which with his mechanical talent he readily succeeded, and afterward went to Auburn to perfect himself in the art; he remained there until he was about twenty-seven years of age, at which time it became necessary for him to give up the business, on account of his feeble health. He had pursued his business closely for a period of nine years; it became necessary for him to recuperate his health; he traveled for that purpose. During his travels he stopped in Troy, N. Y., and passing a window, saw some small tools, similar to those he had been accustomed to use; curiosity led him to enter and inspect them, he discovered to his surprise he was in the office of a dentist, a Dr. Brockway; after due investigation he came to the conclusion that he would be pleased to follow this new business, and thought that with his familiarity in the use of tools he could readily learn and practice the art. His health having improved he at once made an arrangement and entered upon his studentship; he remained with Dr. Brockway but a short time before commencing to operate for himself. His first practice was in Pittsfield, Mass., and Schenectady, N. Y., remaining in the latter place one winter. He then came to Utica, N. Y., where Dr. E. Gidney, an English dentist of some note, was then practicing for a short time. As it was his custom to make trips to Utica occasionally, he at once made an arrangement with Dr. G. to remain with him awhile and receive instruction; in the course of a year he purchased of Dr. G. all his instruments and for a consideration bound him not to practice again in that city. Thus Dr. Blakesley located in Utica about the year 1826, the first dentist, (as he often remarked) that settled permanently west of Albany, where he remained up to the time of his death, a period of thirty-four years, practicing most of the time. The close confinement of practice at times wore upon him so that he was obliged to leave home for a change of climate to recruit his health, and accordingly we find him at various times in Pittsburg, Cleveland, and New Orleans, accompanied always with his favorite instruments with which to work when able. Such was his health in 1845 that by the advice of his physician, he made a trip to Nassau, W. I., and afterwards in 1849, to Hamilton, C. W., practicing in those places when his health permitted. Since that time he has remained at home, hard at work, with but little cessation.

During almost the entire period of his professional life he has labored under the disadvantage of an impaired state of health; but with persistless and untiring energy, he pursued his favorite work not only as a duty but as a pleasure, with the ambition and energy of a young man to the last year of his life; but few men would have stood to the post of duty under like circumstances so faithfully. The past winter and spring, he again showed signs of declining health and great depression of spirits, and by the advice of his physician and friends he consented to take a trip to Savannah for a few weeks; he once more packed up his instruments, intending to practice, (health permitting,) and left Utica, April 14, 1860, never more to return.

Dr. Blakesley was emphatically an honest, upright, honorable dentist, one who prided himself in always doing for his patients the best work that could be done.

Educated by a large experience, he was a thoroughly practical man, one who could demonstrate better than theorize. In all his professional intercourse he endeavored to maintain and advance the dignity and standing of the profession he followed so long and well.

His intercourse with the profession was not very extensive till within a few years; though he was on the most intimate terms with some of its best members. He despised every thing like quackery; he saw that the dental profession, especially in the early period of its history, was cursed with this incubus; this was probably the chief cause of his isolation from the mass of the profession. He attended the meeting of the American Dental Convention at Boston, three years ago; he was delighted to find that the profession was being rapidly elevated; he entered into the schemes and operations of the Convention with as much ardor as the most zealous; he made many valuable suggestions, in regard to the operations of the Convention; since that time he has usually attended the meetings of the profession, and entered with good will into everything that was calculated to promote dental science. As a friend he was ever warm and confiding, while for those who were not his friends he had nought but kind words. In these respects, as well as many others, he was worthy of imitation. The remembrance of him will be green as long as those last who knew him.

